# *WRN *polymorphisms affect expression levels of plasminogen activator inhibitor type 1 in cultured fibroblasts

**DOI:** 10.1186/1471-2261-8-5

**Published:** 2008-02-29

**Authors:** Elena Castro, Vladimir Oviedo-Rodríguez, Luis I Angel-Chávez

**Affiliations:** 1 Centro Universitario de Investigaciones Biomédicas, Universidad de Colima, Colima, México; 2 Facultad de Medicina, Universidad de Colima, Colima, México

## Abstract

**Background:**

Recessive mutations in *WRN *gene eliminate WRN protein function (helicase) and cause Werner syndrome. One of the most important clinical features of Werner syndrome patients are the premature onset and accelerated atherosclerosis process. Studies carried out on polymorphic *WRN *locus have shown that the alleles 1367R and 1074L confer protection for cardiovascular disease. Given that the levels of plasminogen activator inhibitor type 1 (PAI-1) were found to be significantly increased in Werner syndrome patients, is quiet possible that *PAI-1 *expression could be under regulation of WRN helicase. Therefore the purpose of this work was to evaluate the role of *WRN *polymorphism in modulating the expression of *PAI-1*.

**Methods:**

In order to accomplish our aim, an array of primary cultured fibroblasts from normal adult donors was genotyped for polymorphisms of both the *WRN *and *PAI-1 *loci. In addition, steady state levels of *WRN *and *PAI-1 *were measured by semi-quantitative RT-PCR assays in such cultures. To search for the potential relationship between the lack of WRN protein and *PAI-1 *expression, heterozygous cultures of fibroblasts (1367RC/1074LF; *WRN *genotype) were treated with a molecule of interference RNA against *WRN *messenger RNA (mRNA).

**Results:**

We found that, carriers of 1367R and 1074L alleles of *WRN *shown to have low amounts of PAI-1 in plasma (7.56 ± 5.02), as compared with carriers of 1367C and 1074F alleles (16.09 ± 6.03). Moreover, fibroblasts from carriers with these alleles had low expression levels of *PAI-1 *mRNA. The treatment of heterozygous primary fibroblast cultures (1367RC/1074LF; *WRN *genotype) with iRNA against *WRN *mRNA caused *PAI-1 *overexpression. Treatment with normal PAI-1 inducers (TGFβ, TNFα, or insulin) in these cultures and from those with genotypes 1367CC/1074FF and 1367RR/1074FL resulted in a genotype-dependent *PAI-1 *expression level.

**Conclusion:**

Our results suggest that polymorphisms in the *WRN *gene might have a significant role regulating PAI-1 levels in healthy individuals and "normal states" as well as acute or chronic stress, obesity, aging, acute inflammation, among others, where characteristic high levels of insulin, TNF α and TGFβ, could favor PAI-1 high levels in carriers with polymorphic variants (C and F alleles), beyond the levels reached by carriers with other alleles (R and L alleles).

## Background

Werner syndrome (WS) is caused by the inheritance of two copies of null mutations in the *WRN *locus located at chromosome 8 [1]. *WRN *product is a helicase involved in DNA replication and proofreading processes; its mutations eliminate WRN protein function [2], mostly affecting DNA repair [3]. WS is characterized by premature onset and accelerated progression of age-related diseases [4]. From these, vascular pathologies that include atherosclerosis, arteriosclerosis, medial calcinosis, and calcification of heart valves are the major WS pathological components. Indeed, myocardial infarction (and cancer) is the most frequent cause of death in WS patients, which occurs at a median age of 48 years old [4,5]. One of the first evidences that links WRN to the atherosclerosis process during normal aging involves the well known *WRN *polymorphisms: C1367R (refSNP ID: rs1346044) and L1074F (refSNP ID: rs2725362). From these studies it was suggested that variant 1367C is associated with an increased risk of myocardial infarction, whereas variant 1074F is linked to coronary stenosis [6,7]. Moreover, it has been suggested that 1367R allele of the *WRN *gene protects against the development of type 2 diabetes mellitus [8]. Therefore, we hypothesize that WRN protein could be relevant for the regulation of the atherosclerosis process, even in humans without WS. To verify this hypothesis at the molecular level, we studied the expression of plasminogen activator inhibitor type I (PAI-1) in sub-epithelial fibroblasts from humans with differential *WRN *polymorphisms C1367R and L1074F. We focused on PAI-1 because: a) PAI-1 plays a key role in fibrinolysis, thereby modulating the rate of atherogenesis [9] and, b) *PAI-1 *overexpression has been observed both in plasma and fibroblasts from WS patients [10]. To accomplish this objective, an array of primary cultured fibroblasts from healthy Mexican adults was genotyped for polymorphism at the *WRN *locus. Moreover, a semi-quantitative RT-PCR analysis was used to measure the basal level of *PAI-1 *mRNA and the changes elicited by physiological PAI-1-inducers (TNFα, TGFβ, or insulin) when the expression of *WRN *from heterozygous 1367CR/1074LF- fibroblasts was blocked by a molecule of interference RNA (iRNA). We also studied the effect of inducers on fibroblasts with different genotypes. We found that *WRN *polymorphisms have a significant contribution to determine levels of *PAI-1 *expression.

## Methods

### Subjects

Protocol design was approved by the Ethical Committee from University of Colima (No. 2006/54); this protocol is in compliance with the Helsinki Declaration.

One hundred and fifty healthy non-smoking Mexican male subjects were recruited by advertisement. Volunteers had no clinical history of hypertension, diabetes or inflammatory disorders. Haematological and biochemical tests, including white blood cell count (WBC) and C-reactive protein (CRP) were determined to reject subjects with sub clinical inflammation; written informed consent was obtained from each participant after the procedures had been fully explained. Thus, samples from subjects having a BMI lower than 18.5 Kg/m^2 ^or higher than 24.9 Kg/m^2 ^were excluded from this study. Blood samples of volunteers were taken in the morning to avoid normal circadian variation in proteins concentrations [11].

PAI-1 concentration was determined by using HPAIKT kit (Stratech Scientific, Suffolk, England) and following the manufacturer instructions.

### Samples

Explants of dermis (2 mm^3^) with attached epidermis were aseptically removed from donors. Subsequently, tissue was enzymatically dissociated by 5-min incubation with 0.05% trypsin, prepared in phosphate buffer saline (PBS). Fibroblasts were harvested, then plated and maintained in DMEM (Life Technologies, Grand Island, NY) plus 10% fetal calf serum (FCS; Life Technologies, Grand Island, NY), at 37°C in 5% CO_2 _atmosphere. In order to obtain fibroblasts with their cellular cycles synchronized, cultures were grown to confluence and then serum-starved for 72 h (culture medium containing 0.5% serum). Thereafter, cells were re-fed (DMEM + 10% FCS) and twenty four hours later total RNA and DNA were isolated from cells.

### PAI-1 and WRN genotyping

C1367R and L1074F *WRN *polymorphisms and *PAI-1 *promotor polymorphism -675 4G/5G were determined by automatic sequencing. Polymorphic fragments of *WRN *and *PAI-1 *were amplified from genomic DNA by polymerase chain reaction (PCR) in a cycling instrument iCycler (Bio-Rad, Hercules, CA), with the following primers: CR1367F: GCCTAATCAGAATGTTAGTT and CR1367R: CCTCAGTATTGATGCCTACTTC; LF1074F: CTTGTGAAGAGGCCTATAAACTGG and LF1074R: GCCTCATCCTTCAAGCTAATGCAG; PAIF: TACCATGGTAACCCCTGGTC and PAIR: GGATCTGTTAGTGCACCGAC. Sequencing reactions were performed with a Big Dye Terminator V3.1 Sequencing kit (Applied Biosystems, Foster City, CA) following the manufacturer instructions, purified by DyeEx Spin kit (QIAGEN, Valencia, CA) and automatically sequenced by ABI PRISM 310 Genetic Analyzer (Applied Biosystems, Foster City, CA).

### Semiquantitative RT-PCR determination of PAI-1 and WRN expression

Confluent layers of skin fibroblasts were used for RNA isolation. Total RNA was isolated by Trizol Reagent (Gibco BRL, New York, NY). RNA was used to perform a semiquantitative RT-PCR analysis for *PAI-1 *and *WRN *expression For each sample 1.0 μg RNA was reverse transcribed and amplified by Superscript One-Step RT-PCR kit (Invitrogen, Carlsbad, CA) following the manufacturer's instructions using the following set of intron spanning primers, annealed into the coding sequence in order to determine splicing variants [12]: ActinF: CTCGTCGTCGACAACCGGCT, ActinR: GCCAATGGTGATGACCTGGC, PAIF: CAAGGATGAGATCAGCACCACAG, PAIR: GGGTTCCATCACTTGGCCCATG WRNF: TGACTTCAATCTCTGAGGAAG and WRNR: CAGCTCTACCAATCTCCTGA. PCR products were separated in an agarose gel (2%) electrophoresis and stained with ethidium bromide. The intensity of the fluorescence was automatically measured and integrated by gel data acquisition "Gel Doc" (Bio-Rad, Hercules, CA) software. All RT-PCR experiments were done in triplicate and 26 amplification cycles were used.

### iRNA preparation

The iRNA sequences were as follows: sense UAGUGGCACGGUAGAACCAdTT, antisense UUGUUCUACCGUGCCACUdTT. A scrambled sequence was designed as a mismatch control: sense GUCACACGAUAGCGAGGUAdTT and antisense UACCUCGCUAUCGUGUGACdTT. These RNA oligonucleotides were synthesized individually, deprotected and purified by RNase-free HPLC (Invitrogen, Carlsbad, CA). iRNA and mismatch RNA (scrambled) duplexes were formed following the manufacturer instructions.

### Transfection with iRNA against WRN messenger

Five fibroblast cultures presenting heterozygosis for C1367R *WRN *and for F1074L *WRN *(1367CR/1074FL genotype) were synchronized and re-fed (DMEM/10%SFC), to be immediately used for iRNA treatment. Cells were transfected with 2 μg siRNA/i-Fect complexes (Invitrogen, Carlsbad, CA) in a 1:4 ratio (w/v) per plate. Twenty four hours after transfection, cells were treated with TNFα (150U/mL; Genzyme. Cambridge, MA), TGFβ (18U/mL; Genzyme. Cambridge, MA) or insulin (1 mg/mL; Sigma. St. Louis, MO). Twenty four hours later, cells were harvested and total RNA was isolated. *PAI-1 *and *WRN *expression was evaluated by RT-PCR. The same procedure was followed for scrambled RNA and control.

Fibroblast cultures with genotypes 1367CC/1074FF and 1367RR/1074FL were treated equally, except for iRNA treatment. Five cultures for each genotype were used.

### Western blot

Cultured fibroblasts treated and untreated with iRNA or scrambled molecule were lysed with sodium dodecyl sulfate (SDS) sample buffer. Proteins were quantified using the Bradford assay reagent. Equal amounts of protein (50 μg) were separated under denaturing conditions, by SDS-PAGE on 10–20% polyacrylamide Tris-glycine gels, and then electroblotted to polyvinylidene difluoride (PVDF) membrane. Non-specific sites were blocked with 5% non fat dry milk in 0.2% Tween-20 in Tris-buffered saline (TBS-T) for 1 hour at room temperature then reacted with the primaries anti-WRN and anti PAI-1 antibodies for 1 hour, followed by horseradish peroxidase (HRP)-labeled anti-rabbit IgG (Jackson Laboratories) for 1 hour. The bands were visualized using an ECL Plus kit (Amersham Pharmacia Biotech, Amersham, UK). β-actin was detected as a protein loading control in the same membrane.

### Statistical Evaluation

Values are presented as mean ± SD or mean ± SEM. The difference of PAI-1 levels between genotypes was analyzed by paired Student's test. One-way ANOVA was used to test differences in *PAI-1 *expression trough *WRN *genotypes and in *WRN *knocking down experiments across treatments (control, scrambled and iRNA). Significant results from ANOVA were further analyzed by Tukey's post hoc test. P < 0.05 was considered significant.

## Results

### Studied population with no evidence of inflammation

PAI-1 is commonly present at low levels in plasma form healthy humans; however, acute and chronic diseases are strongly associated with increased *PAI-1 *expression and release [13]. Therefore, to avoid that PAI-1 quantification was not influenced by sub-clinical inflammation, different serum markers of inflammation were measured as well, thereby trying to avoid high or low levels of PAI-1 independently of *WRN *genotype. Thus, plasmatic concentration values of C-reactive protein (CRP), an acute phase protein, fibrinogen, glucose, creatinine (through others markers) are shown in Table 1. Such normal values demonstrate that the studied population (n = 150) did not have evidence of inflammatory processes.

**Table 1:** Biochemical and haematological characteristics of the studied population

	Mean ± SD		Mean ± SD
Age	45.4 ± 4.4	Fibrinogen (mg/dl)	207.61 ± 33.2
BMI (kg/m^2^)	23.4 ± 1.2	CRP (mg/l)	0.8 ± 0.16
Glucose (mg/dl)	93.7 ± 7.81	RBC (10^6^/mm^3^)	4.721 ± 0.98
Urea (mg/dl)	8.86 ± 3.02	Haemoglobin (g/dl)	12.9 ± 2.45
Creatinine (mg/dl)	0.92 ± 0.07	Haematocrit (%)	45.73 ± 2.67
Albumin (mg/dl)	4.23 ± 0.36	WBC (10^6^/mm^3^)	0.71 ± 0.21
Cholesterol (mg/dl)	150 ± 24	Platelets (10^6^/mm^3^)	28.67 ± 6.66
Triglycerides (mg/dl)	102 ± 43	PAI-1 (ng/ml)	9.73 ± 6.72

N = 150 individuals. SD = Standard deviation, BMI = Body mass index, CRP = C reactive protein, RBC = red blood cells, WBC = white blood cells, PAI-1 = plasminogen activator inhibitor type 1

### The amount of PAI-1 in plasma is dependent of the *WRN *genotype

In order to know whether the amount of PAI-1 in plasma is related of *WRN *genotype, PAI-1 concentration was determined as described in methods. Subsequently, *WRN *polymorphisms (rs1346044 and rs2725362) were evaluated from genomic DNA. Table 2 summarizes the amount of PAI-1 from a Mexican male population endowed with the following *WRN *genotypes: 1367CC/1074FF, a genotype whose alleles are not related to cardiovascular disease protection and 1367 CR/1074LL, 1367 RR/1074FL or 1367 RR/1074LL, whose alleles are associated with protection against cardiovascular disease [6,7]. It was found that the individuals with 1367CC/1074FF genotype systematically revealed higher levels of PAI-1 as compared with those with genotypes whose alleles were associated with protection to cardiovascular disease (P = 0.031).

**Table 2:** Biochemical and haematological characteristics of individual with differential genotype of *WRN*

**Genotype of *WRN***	**1367CC/1074FF**	**1367CR/1074LL** **1367RR/1074FL** **1367RR/1074LL**	P value
n	25	12	

Fibrinogen (mg/dl)	200.92 ± 7.04	204.81 ± 8.9	0.077
CRP (mg/l)	0.8 ± 0.012	0.8 ± 0.063	0.5
RBC (10^6^/mm^3^)	4.832 ± 0.206	4.543 ± 0.24	0.139
Creatinine (mg/dl)	0.92 ± 0.056	0.93 ± 0.026	0.458
WBC (10^6^/mm^3^)	0.69 ± 0.008	0.73 ± 0.05	0.283
PAI-1 (ng/ml)	16.09 ± 1.206	7.56 ± 1.45	**0.031**

Values are shown as mean ± standard error of the mean. CRP = C reactive protein, RBC = red blood cells, WBC = white blood cells, PAI-1 = plasminogen activator inhibitor type 1. The differences between groups were analyzed by paired Student's test

### Dependence of the basal level of *PAI-1 *expression on the *WRN *genotype

**Figure 1:**
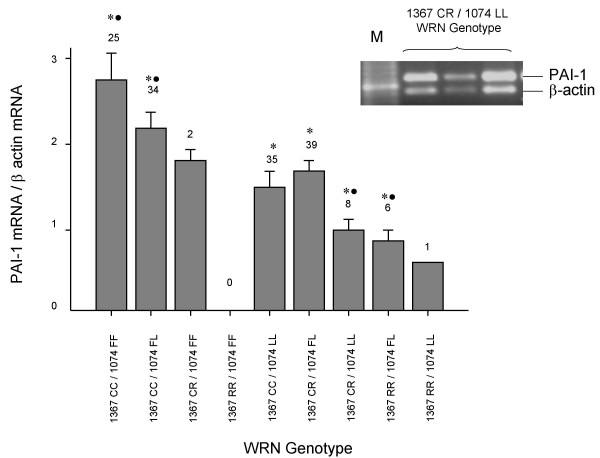
***PAI-1 *steady state levels in primary cultures of fibroblasts with several *WRN *genotypes**. *WRN *C1367R and L1074F polymorphisms were determined by automatic sequencing in primary cultured fibroblasts obtained from healthy adult donors; steady state levels of *PAI-1 *were determined by semiquantitative RT-PCR after 26 amplification cycles. One representative agarose gel is shown in inset. Results were normalized to β-actin expression and plotted as mean ± standard deviation of arbitrary units. Number of cultures from each genotype is shown above each bar. * Groups used for ANOVA test • Groups with statistic differences against heterozygous carriers (1367CR/1704FL) by Tukey test, P < 0.01. M = molecular weight ladder.

To address whether *WRN *polymorphisms can modulate *PAI-1 *expression, a semiquantitative RT-PCR analysis of *PAI-1 *gene expression was done in an array of primary cultured fibroblasts obtained from the same Mexican male population. Constant amounts of reverse-transcribed mRNA were amplified together with the constitutive gene β-actin to normalize *PAI-1 *expression. Figure 1 shows that fibroblasts from carriers 1367RR and/or 1074LL expressed the lowest *PAI-1 *levels, whereas those carrying the 1367CC and/or 1074FF alleles expressed the highest ones. These results suggest that WRN may modulate *PAI-1 *expression or, alternatively, its differential expression may depend on *PAI-1*-promoter polymorphisms such as -675 4G/5G [14]. To evaluate the latter possibility, fibroblasts used for the above experiments were further genotyped for -675 4G/5G. There were no significant differences in the steady-state level of *PAI-1 *among groups with different *PAI-1 *genotypes (data no shown).

### Effect of the transient knock-down of *WRN *on *PAI-1 *expression

**Figure 2:**
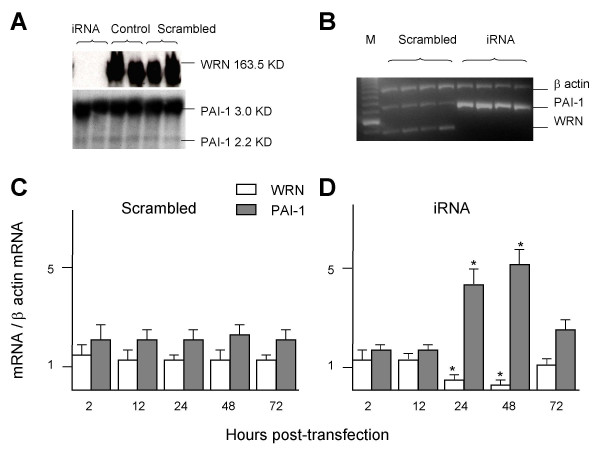
***PAI-1 *expression levels in *WRN *knocked down fibroblasts**. Five synchronized fibroblast cultures presenting heterozygosis for *WRN *(1367CR/1074LF) were transfected with a molecule of iRNA against *WRN *messenger (iRNA; panel D), or with a scrambled control RNA (Scrambled; panel C). *PAI-1 *and *WRN *expression was evaluated by semiquantitative RT-PCR at several times post-transfection. Normalized values of mRNA were grouped as mean ± standard error of arbitrary units. ANOVA was used to test differences across treatments (control, scrambled and iRNA). * Significant differences compared with scrambled by Tukey test; P < 0.01. Cells cultures were subjected to immunoblotting with anti-WRN antibody to monitor *WRN *knocking down (panel A). Semiquantitative RT-PCR was done at 26th cycle of amplification from total RNA isolated from cell cultures, products were separated by electrophoresis in 2% agarose gel and stained with ethidium bromide. The intensity of the fluorescence was measured and integrated by "Gel Doc" software. All RT-PCR experiments were done by triplicated (panel B) M = molecular weight ladder.

To search for the potential regulatory role of WRN in *PAI-1 *expression, five cultures of heterozygous fibroblasts carrying the *WRN *genotype, 1367CR/1074LF, were transfected with a specific iRNA against *WRN *mRNA to transiently block its expression. The effectiveness of *WRN *silencing was evaluated by western blot analysis for each cell culture, as shown in the autoradiography in Figure 2A. All of those cell cultures treated with iRNA against *WRN *messenger, were not reactive (or scarcely reactive) to the antibody against WRN. In contrast, cell cultures treated or not with the scrambled molecule presented a high reactive signal (data no shown). Thereafter, mRNA levels of *WRN *and *PAI-1 *were measured in those knocked-down cultures. As expected, fibroblasts showed significant reduced levels of *WRN *mRNA at 24 and 48 h post-transfection (open bars in Figure 2D). During the same period there was a parallel large enhancement of *PAI-1 *expression that reached a maximum of ~400 % 48 h upon transfection with the iRNA (filled bars in Figure 2D). The effect of the iRNA on levels of *WRN *mRNA and *PAI-1 *mRNA were reversed after 72 h post-transfection. In contrast, the expression of neither *WRN *nor *PAI-1 *was modified by the scrambled control RNA molecule (Figure 2C). Moreover, there were no significant differences in *WRN *and *PAI-1 *expression between treated and untreated fibroblasts with the scrambled RNA molecule (data not shown). Taken together, these results suggest that WRN tightly regulates *PAI-1 *expression.

### Effect of PAI-1-inducers on *PAI-1 *overexpression in fibroblasts treated with iRNA against *WRN*

**Figure 3:**
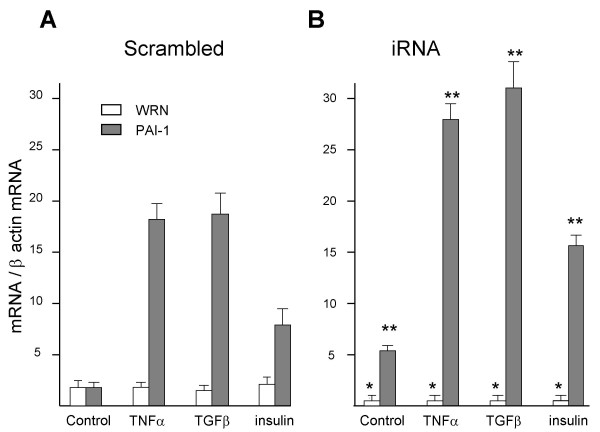
***PAI-1 *expression levels in response to TNFα, TGFβ, or insulin in WRN knocked down fibroblasts**. Five synchronized heterozygous fibroblasts cultures (1367CR/1074LF) were transfected with a molecule of iRNA against *WRN *messenger (B), or with a scrambled RNA as control (A). PAI-1 inducers (TNFα, TGFβ, or insulin) were added 24 hours post-transfection and *PAI-1 *and *WRN *expression was evaluated by semiquantitative RT-PCR 24 hours after agonist challenge. Normalized values of mRNA were grouped as mean ± standard error of arbitrary units. ANOVA was used to test differences across treatments (control, scrambled and iRNA). * Significant differences compared with scrambled by Tukey test; P < 0.05 and ** P < 0.01.

It is well known that signaling molecules including TNFα, TGFβ and insulin are inducers of *PAI-1 *expression [15,16]. To search for the potential cross-talk between the WRN-dependent regulation of *PAI-1 *expression and the signaling pathways used by those physiological PAI-1-inducers, TNFα (TGFβ, or insulin) was added 24 hours after heterozygous fibroblasts (1367 CR/1074LF) were transfected with iRNA against *WRN*. *PAI-1 *and *WRN *expression was measured 24 hours after the addition of inducers to coincide their maximum effect (24 h) with that of iRNA (48 h). In cells transfected with the scrambled molecule, TNFα (TGFβ, or insulin) produced a large increase of *PAI-1 *expression (Figure 3A, filled bars) without any noticeable effect on expression of *WRN *(Figure 3A, open bars). As expected, in fibroblasts transfected with the iRNA, there was a reduction in *WRN *mRNA (Figure 3B, open bars) and in that condition, TNFα, TGFβ, or insulin produced a further overexpression of *PAI-1 *(Figure 3B, filled bars). *WRN *expression showed no significant differences among treatments, neither in the scrambled nor in iRNA transfected cells (Figure 3A and 3B). Moreover, there were no significant differences in *WRN *and *PAI-1 *expression between treated and untreated fibroblasts with the scrambled RNA molecule (data not shown).

These results confirm that WRN controls *PAI-1 *overexpression and suggest that when *WRN *is knocked down, *PAI-1 *escapes this control and can be over-induced by TNFα, TGFβ, or insulin (Figure 3B). Experimental inducer concentrations (TNFα, TGFβ and insulin) were previously standardized at 150 U/ml, 18 U/ml and 1 mg/ml, respectively. Higher concentrations did not significantly augment *PAI-1 *expression when they were added to the culture medium in normal fibroblast cultures (data not shown).

### *Relation between WRN *polymorphisms and *PAI- 1 *expression induced by TNFα, TGFβ or insulin

**Figure 4:**
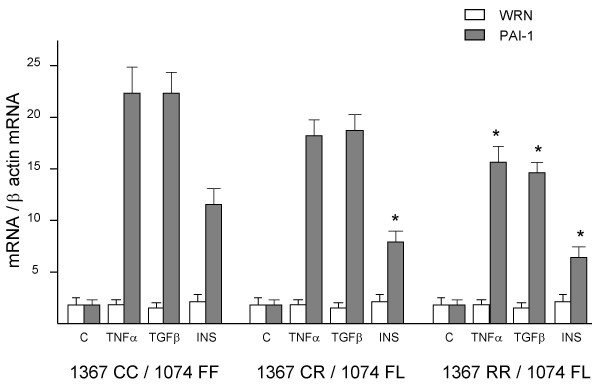
***PAI-1 *induced levels in fibroblasts with different *WRN *genotypes**. Synchronized fibroblasts with different *WRN *genotypes were treated with TNFα, TGFβ, or insulin. Twenty four hours later, *PAI-1 *and *WRN *expression was evaluated by semiquantitative RT-PCR. Expression results from five cultures were grouped as mean ± standard error of arbitrary units, normalized to β-actin expression. ANOVA test was used to determine differences across genotypes * P < 0.01 against 1367CC/1074FF genotype by Tukey post hoc test.

Given that *WRN *polymorphisms may modulate constitutive *PAI-1 *expression (Figure 1), we examined whether *WRN *polymorphisms could affect *PAI-1 *overexpression induced by TNFα, TGFβ, or insulin. Therefore, we used fibroblasts from individuals with the following genotypes: 1367CC/1074FF, 1367CR/1074FL and 1367RR/1074FL (we used the last genotype instead of 1367RR/1074LL, because we had just one culture from an individual with that genotype). Cultures were synchronized and re-fed and they were immediately treated with PAI-1 inducers and 24 hours later, cells were used to determine *WRN *and *PAI-1 *expression levels. TNFα, TGFβ, or insulin elicited significant lower expression of *PAI-1 *in fibroblasts with the 1367RR/1074FL genotype compared with that found in cells with the 1367CC/1074FF genotype (Figure 4). Insulin-dependent overexpression *PAI-1 *gave also significant differences in fibroblasts with the1367CR/1074FL genotype. We did not find significant differences when comparing other groups.

## Discussion

We have demonstrated a clear role of WRN in regulating *PAI-1 *expression because primary cultured fibroblasts expressed high *PAI-1 *levels when *WRN *mRNA was blocked by a selective iRNA molecule. This molecular finding is in agreement with previous observations showing that WS patients, lacking WRN protein action, exhibit high PAI-1 levels in plasma and their fibroblasts overexpress *PAI-1 *[10]. It is accepted that WS patients have premature and accelerated progression of age-related disorders that resemble those during normal aging [4,17]. Thus, the WRN-dependent *PAI-1 *expression reported here is also in agreement with the fact that both fibroblasts from elderly people or fibroblasts in replicative senescence overexpress *PAI-1 *[17-19], in parallel with a reduction of *WRN *mRNA [20].

We do not know whether WRN affects *PAI-1 *expression by inhibiting its RNA production or by blocking some signaling pathway. We favor the former mechanism because the transcription efficacy in cells from WS individuals is reduced to 40–60% as compared with the rate of transcription in cells from normal subjects [21], suggesting that WRN is implicated in regulating transcription process. Further investigation is needed to clarify the precise relationship between WRN helicase and *PAI-1 *mRNA.

The polymorphisms studied here are located in the C-terminal region of WRN which contains exonuclease [22], helicase [23,24] and transactivation activities [6,21], besides the nuclear localization signal. This region serves as a binding site for interacting proteins and DNA [25]. The C1367R polymorphism is close to the sequence coding the nuclear localization signal and adds a basic amino acid in this region (coded by the R allele), which it has been suggested that enhances the strength of the nuclear localization [6]. In other hand, the L1074F polymorphism is close to the DNA-binding domain and introduces an aliphatic amino acid (coded by the L allele) which might influence the transcription efficiency or the interaction with DNA or other factors [6]. In this context, our results show that *WRN *genetic background seems to influence the level of *PAI-1 *expression because fibroblasts from carriers with 1367RR/1074LL *WRN *genotype expressed the lowest *PAI-1 *mRNA levels, whereas fibroblasts from those carriers with 1367CC/1074FF genotype expressed the highest ones. In agreement with *PAI-1 *mRNA data, it was found that the levels of PAI-1 in plasma had a variability that was according to *WRN *polymorphisms. This variation was independent of inflammation as long as participants showed normal values of fibrinogen, WBC and CRP among other inflammation markers. Taken together, these findings show that polymorphisms in *WRN *gene differentially contribute to the levels of *PAI-1 *expression, a regulation that was experimentally revealed upon the disruption of the *WRN *expression. Therefore, in healthy individuals WRN may have clinical significance because polymorphic variants (C and F alleles) may be prone to increased risk of atherosclerosis due to their higher PAI-1 levels.

In this study we also found that TNFα, TGFβ, or insulin produced a further *PAI-1 *expression even so *WRN *knocked down enhanced it already. This additive effect was quite expected due to *PAI-1 *expression was negatively and positively regulated by WRN and the PAI-1-inducers, respectively. The agonist-dependent enhancement of *PAI-1 *overexpression was also affected by *WRN *polymorphisms. That is, polymorphic variants (C and F alleles) promoting higher PAI-1 levels in plasma and *PAI-1 *mRNA also supported a large overexpression of *PAI-1 *by insulin, TNFα and TGFβ. *WRN *polymorphisms may also have relevant clinical significance for "normal states" such as stress, obesity, aging or acute inflammation, because they are characterized by high levels of insulin, TNFα, TGFβ [26-29]. Therefore, carriers with some polymorphic variants (C and F alleles) will favour PAI-1 higher levels than those reached by carriers with other alleles (R and L alleles). Thus, C and F polymorphic variants may imply and enhanced risk of suffering various diseases, such as atherosclerosis [30,31], cancer [32,33], diabetes [34,35], wound healing failure [36,37], among others.

The WRN-dependent regulation of *PAI-1 *expression described in fibroblasts opens an avenue to address whether a similar effect occurs in other cellular types that have a greater impact in the systemic amount of PAI-1, such as vascular smooth muscle, macrophages and endothelial cells. This potential widespread up-regulation of *PAI-1 *expression could be quite relevant for atherosclerosis because it begins as a response to endothelial injury, and one of the earlier events consist of fibroblasts proliferation and differentiation at the level of the intimal connective tissue. In this layer of the vascular wall, PAI-1 acts as an inhibitor of the extracellular matrix degradation and it has been shown that PAI-1 is a critical mediador of intimal growth [38].

Therefore, the up-regulation of *PAI-1 *from fibroblasts may play an important role in the molecular pathogenesis of fibrosis during the atherosclerosis process. Further work is required analyzing intracellular signalling and promoter regions implicated in the *PAI-1 *up-regulation WRN-dependent.

## Conclusion

We conclude that in fibroblasts the WRN protein down-regulates *PAI-1 *expression and after knocking down *WRN *gene, *PAI-1 *expression is enhanced. Our results suggest that polymorphisms in *WRN *gene significantly contribute to regulate the levels of *PAI-1 *expression. In consequence, *WRN *polymorphisms may have a relevant clinical significance in healthy individuals. Since, polymorphic variants (C and F alleles) favor higher PAI-1 levels and also overexpress *PAI-1 *in response to insulin, TNFα and TGFβ; it may imply an enhanced risk of suffering various conditions, such as atherosclerosis, cancer, diabetes, wound healing failure, among other diseases.

## Competing interests

The author(s) declare that they have no competing interests.

## Authors' contributions

EC conceived the study, participated in its design, and performed the recruitment of subjects, establishment of primary cultures, *WRN *and *PAI-1 *genotyping, and RT-PCR assays, as well as carried out the immunoassays and drafted the manuscript. VO carried out the iRNA assays and the treatment of cell cultures with PAI-1 inducers. LIA participated in the design of the study, performed haematological and biochemical tests and the statistical analysis. All authors read and approved the final manuscript.

## Pre-publication history

The pre-publication history for this paper can be accessed here:


